# A reproducible semi-automatic method to quantify the muscle-lipid distribution in clinical 3D CT images of the thigh

**DOI:** 10.1371/journal.pone.0175174

**Published:** 2017-04-28

**Authors:** Alexander Mühlberg, Oleg Museyko, Jean-Denis Laredo, Klaus Engelke

**Affiliations:** 1 Institute Of Medical Physics, Friedrich-Alexander University Erlangen-Nuremberg, Erlangen, Germany; 2 AP-HP, Radiologie Ostéo-Articulaire, Hôpital Lariboisière, Université Paris VII Denis Diderot, Paris, France; INIA, SPAIN

## Abstract

Many studies use threshold-based techniques to assess *in vivo* the muscle, bone and adipose tissue distribution of the legs using computed tomography (CT) imaging. More advanced techniques divide the legs into subcutaneous adipose tissue (SAT), anatomical muscle (muscle tissue and adipocytes within the muscle border) and intra- and perimuscular adipose tissue. In addition, a so-called muscle density directly derived from the CT-values is often measured. We introduce a new integrated approach to quantify the muscle-lipid system (MLS) using quantitative CT in patients with sarcopenia or osteoporosis. The analysis targets the thigh as many CT studies of the hip do not include entire legs The framework consists of an anatomic coordinate system, allowing delineation of reproducible volumes of interest, a robust semi-automatic 3D segmentation of the fascia and a comprehensive method to quantify of the muscle and lipid distribution within the fascia. CT density-dependent features are calibrated using subject-specific internal CT values of the SAT and external CT values of an in scan calibration phantom. Robustness of the framework with respect to operator interaction, image noise and calibration was evaluated. Specifically, the impact of inter- and intra-operator reanalysis precision and addition of Gaussian noise to simulate lower radiation exposure on muscle and AT volumes, muscle density and 3D texture features quantifying MLS within the fascia, were analyzed. Existing data of 25 subjects (age: 75.6 ± 8.7) with porous and low-contrast muscle structures were included in the analysis. Intra- and inter-operator reanalysis precision errors were below 1% and mostly comparable to 1% of cohort variation of the corresponding features. Doubling the noise changed most 3D texture features by up to 15% of the cohort variation but did not affect density and volume measurements. The application of the novel technique is easy with acceptable processing time. It can thus be employed for a comprehensive quantification of the muscle-lipid system enabling radiomics approaches to musculoskeletal disorders.

## Introduction

Loss of muscle function, as a result of diseases such as neuropathies and myopathies on one hand, or sarcopenia, the age-related loss of muscle mass and function on the other, results in reduced function. Muscle diseases also play a role in the pathogenesis of osteoporosis, falling incidence and bone frailty and fractures. Muscle biopsy is the gold standard for muscle assessment but is invasive and only evaluates a small sample, not always representative, of the relevant muscle tissue. Two different approaches are currently available for *in vivo* 3D muscle and fat evaluation. The first consists of a qualitative or, at best, semi-quantitative grading of muscle structures based on a washed-out and moth-eaten muscle appearance in MRI [1–3] or CT [4, 5] images. The second approach consists of a quantitative measurement of muscle volume or cross-sectional area (CSA) in both MRI or CT images and density measurements with CT [6–10]

While MRI has advantages in the qualitative assessment of muscle structures due to its superior soft tissue contrast, quantitative MRI analyses are affected by complex artifacts such as proton spin inhomogeneities, which usually depend on the specific MR scanner and acquisition sequence. In contrast, CT is less affected by technical variations [11], and provides a higher spatial resolution, which is an important advantage for fine-grained measurements as 3D texture. In addition, muscle density cannot be measured by MRI.

For a radiomics [12] approach to the muscle-lipid system (MLS), a reproducible, robust and fast segmentation and quantification method is required, to exploit more diagnostic relevant information from CT images. This study specifically targeted the thigh instead of the whole upper or even combined upper and lower legs, because many CT scans originally performed to determine bone mineral density of the hip extend to the upper to mid shaft of the femur.

### Tissue composition of the thigh

**Fig 1:**
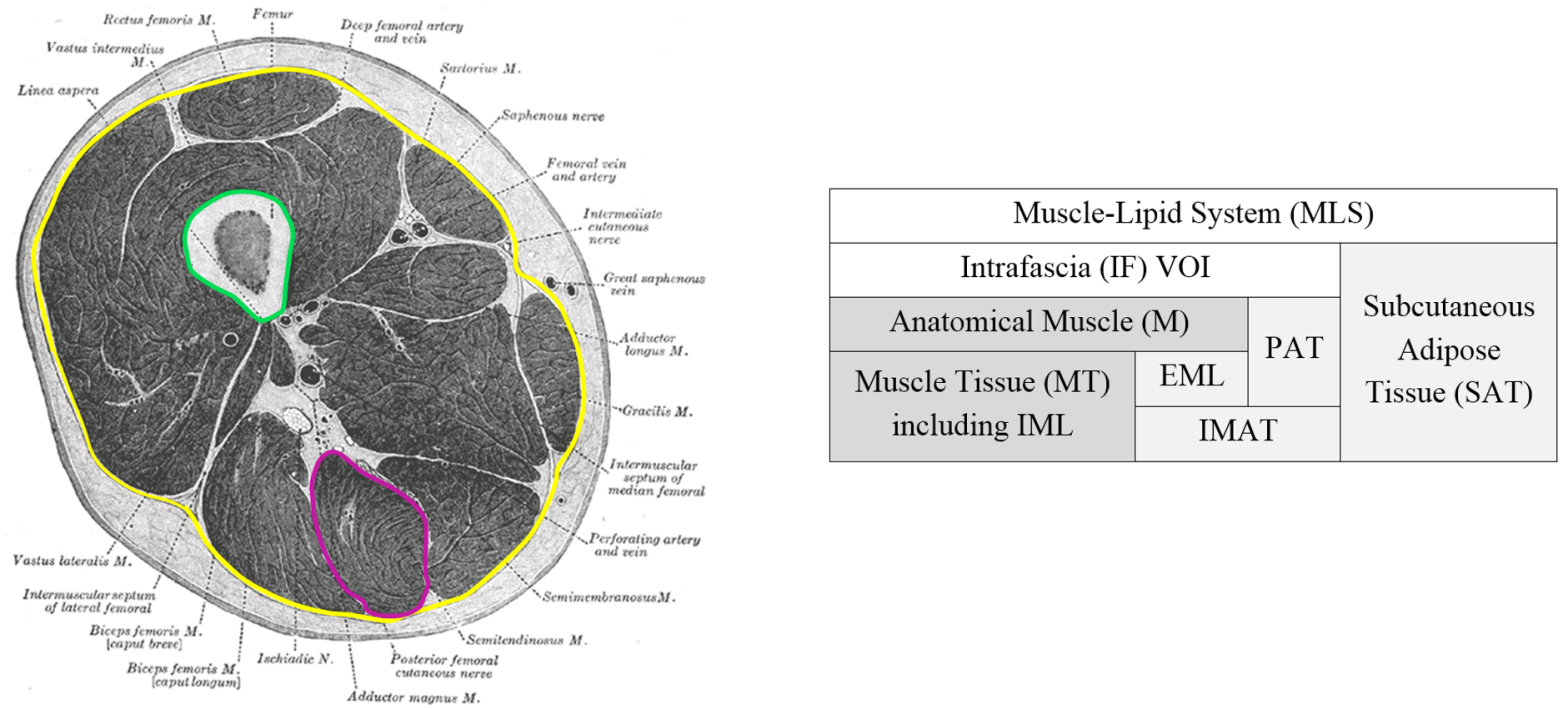
Muscle-lipid system (MLS) of the thigh. Cross section of the femur (left) and schematic composition (right) with muscle (dark grey) and adipose tissue (light grey). The fascia is shown in yellow and the periosteal bone surface in green. AT within the fascia is termed intermuscular AT (IMAT), outside subcutaneous adipose tissue (SAT). IMAT within the anatomical muscle M (magenta contours define the border of the Semitendinosus) is termed extramyocellular lipids (EML) and outside perimuscular AT (PAT). In CT images intramyocellular lipids (IML) can only be measured indirectly by a lower average CT value compared to that of pure muscle tissue.

For a better understanding of the specific segmentation and quantification techniques described in the following sections, a brief overview of the various tissues and compartments of the thigh will be given. The outer layer of the leg is the subcutaneous adipose tissue (SAT) with the skin as an outer surface and the deep fascia (F) as an inner surface (Fig 1). The volume of interest (VOI) inside the fascia (VOI_IF_: IF = Intrafascia) can be separated into the femoral bone, anatomical muscles (M) consisting of muscle tissue and adipocytes within the muscle border and perimuscular adipose tissue (PAT) separating the anatomical muscles. In the present work, no attempt was made to separate individual muscles. Therefore, M will always refer to the combination of all muscles. Adipose tissue (SAT and PAT) consists of adipocytes (fat cells) containing lipids.

Muscle tissue can be further differentiated into myocytes (muscle fibers), which may contain intramyocellular (IML) [13], and extramyocellular lipids (EML). In a CT image, IML can be measured indirectly only via the CT muscle density, which will decrease with increasing IML [14]. EML are adipocytes embedded within M among the muscle fibers. EML is typically present as adipocyte clusters, which, depending on their size and the spatial resolution of the CT image, can be directly segmented because their CT value differs from that of the surrounding muscle tissue. According to reference [15] IMAT (intermuscular AT) will include both PAT and EML in the present study (Fig 1), although some authors [16] refer to PAT only.

## Materials and methods

The integrated approach can be divided into four steps: (A) definition of a global VOI analysis of the thigh, (B) segmentation of the fascia to delineate SAT and IMAT, (C) segmentation of muscle and lipid VOIs within VOI_IF_ and (D) definition of features characterizing the muscle-lipid distribution within VOI_IF_. CT scans of the proximal femur, obtained earlier [17] to measure bone mineral density in subjects with high fracture risk, were used. Subjects were scanned on top of a calibration phantom to calculate BMD from the measured CT values. The femoral bone was segmented as part of a previous study [18].

### A. Analysis VOI

The thigh surface was first segmented using a threshold-based volume growing. The threshold, which was empirically selected as 70%-threshold between highest and lowest CT values, was determined from the CT value spectrum of a sphere automatically positioned on the thigh surface.

**Fig 2:**
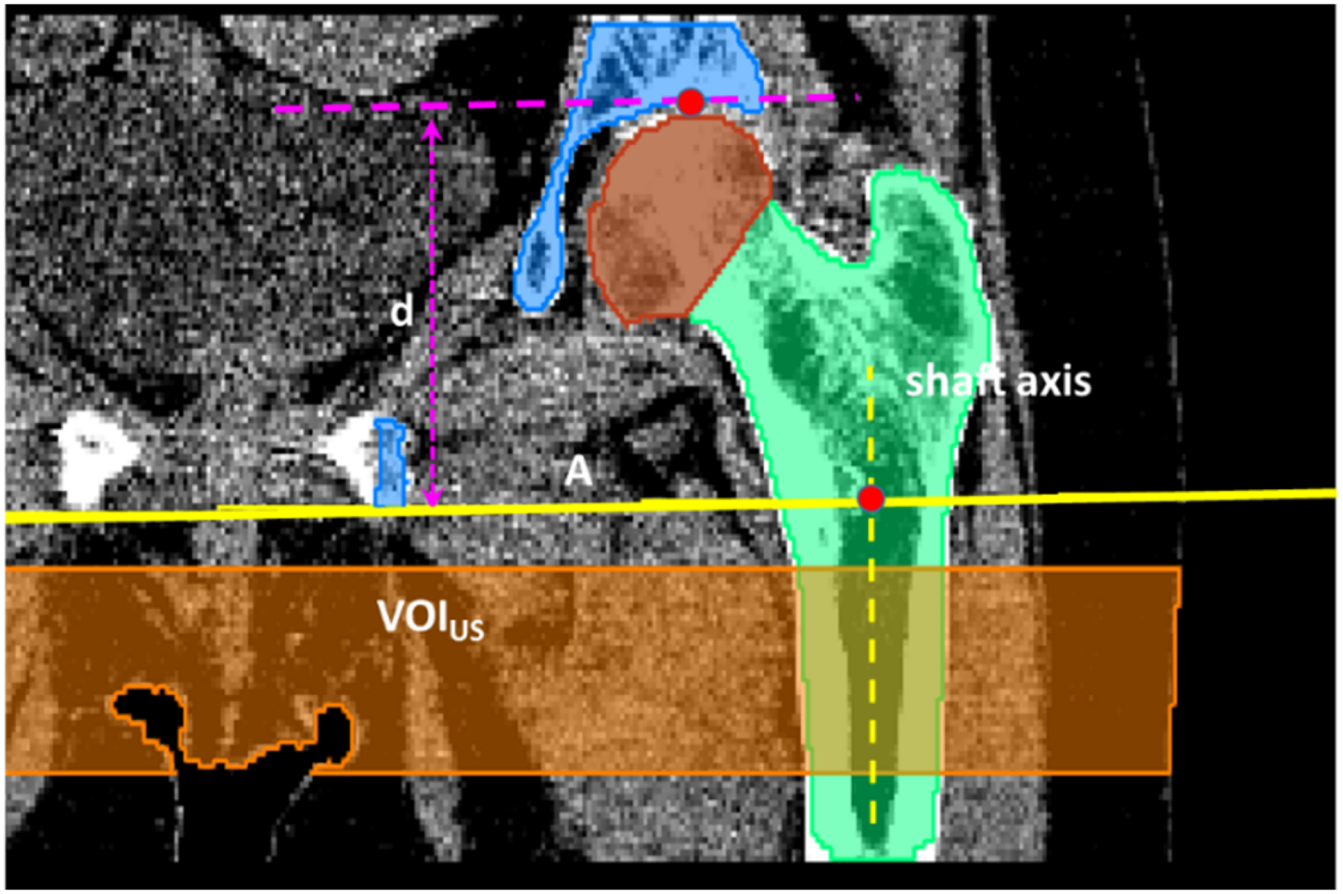
Definition of the analysis VOI. VOI_US_ defined by 1: The center of mass of the femur in plane A, which is perpendicular to the shaft axis and intersects the center of the trochanter minor and 2.: The voxel of the femoral head with maximum distance from A. The projected distance between the two points onto the scanner z-axis is used as an anatomic size- and pose-specific distance d. VOI_US_ contains n = 0.5 d / s (s: slice thickness) slices; the most proximal slice contained the distal end of the acetabulum (not visible here).

The muscle-lipid analysis was carried out at the level of the upper femoral shaft (VOI_US_; Fig 2). Segmentation of the VOI_US_ started with the automatic determination of two points: the first was the center of mass of the femur in plane A, which was perpendicular to the femoral shaft axis and intersected the mid-height of the lesser trochanter. The second point was the voxel of the femoral head at a maximal distance from A. The projected distance between the two points onto the scanner z-axis was used as an anatomic size- and pose-specific distance d. VOI_US_ consisted of n = 0.5 d / s (s: CT slice thickness) slices. The most proximal slice was defined as the distal end of the acetabulum.

### B. Segmentation of the fascia

**Fig 3:**
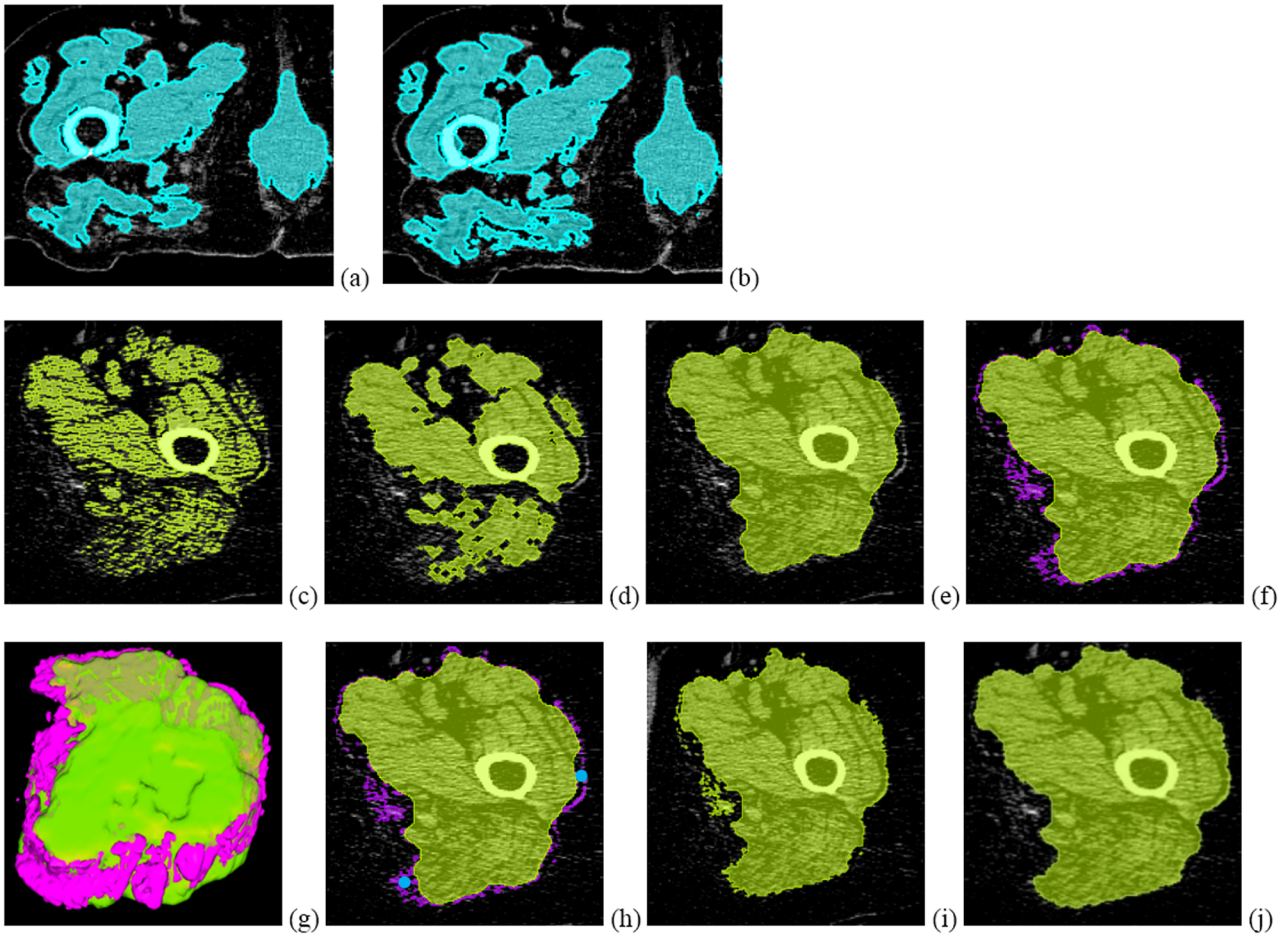
Multi step 3D hierarchical segmentation of the volume of interest inside the fascia (VOI_IF_) exemplified in one 2D axial slice. Step 1: Connected components of right leg (VOI_RC_) for G = 1 (a) and G = 3 (b) (G: Grade of muscle appearance, see text). Step 2: Probable muscle voxels within processed left leg (VOI_LC_) (c) and compactification resulting in VOI_IFA_ (d and e) (IFA: Approximation of IF). Step 3: refinement of VOI_IFA_. Candidate voxels close to FA that may belong to VOI_IF_ (f and g), seed points to select clusters that belong to VOI_IF_ (h), clusters that were not selected stay porous (i) and are removed by morphological smoothing yielding the final result (j). User-dependence is indicated by blue color.

Accurate fascia segmentation is essential for the separation of SAT from muscle and IMAT. However, fascia segmentation is difficult since it is a very thin structure with low contrast relative to the surrounding AT. Pure muscle tissue has higher CT values than AT. However, muscle tissue with high lipid infiltration has CT values close to those of AT. Muscle had also to be distinguished from blood vessels, edema, dermis and genital organs through the following steps:

1. The first step was a gross identification of muscle, based on its CT appearance: a grade G in the range [1, 3] was first set by the operator where G = 3 denotes moth-eaten and washed- out muscle structures (Fig 3a and 3b). In addition, a contrast value (C), relative to AT and water was assigned to each voxel (v) according to a linear scale where C = 0 for a CT value equal to the average CT value of the adipose tissue (CT_AT_) and C = 1 for a CT value equal to that of water. CT_AT_ was determined in a VOI_AT_ resulting from simple volume growing using a CT value range [-190 HU; -30 HU], which started within the sphere on the thigh surface. CT_H2O_ was determined from the in-scan calibration phantom.

In order to classify a voxel v as potential muscle, four empirical conditions had to be fulfilled: (1) a minimum distance of 10 voxels from the body surface; (2) connection with the femur; (3) a contrast C above the minimum contrast C_min_ characterizing the washed-out appearance; and (4) an aggregation α above the minimum aggregation α_min_ characterizing the moth-eaten appearance, where:

$$\alpha \left(\text{v}\right)=\frac{\sum_{{\text{v}}^{\prime }=1}^{26}\theta \left(\text{C}\left({\text{v}}^{\prime }\right)-{\text{C}}_{\text{min}}\right)}{26}$$

$${\text{C}}_{\text{min}}=0.75-0.15\text{ G}$$

$$\alpha_{\text{min}}=0.85-0.05\text{ G}$$

Θ denotes the Heaviside function and v’ is a voxel in the 26-neighborhood of the voxel v. The aggregation value is the ratio of neighboring voxels with the contrast above C_min_.

From the resulting voxel clusters only the two largest connected components within VOI_US_ were kept. According to the position of their center of mass relative to the femur, the two components were classified as right (VOI_RC_) or left (VOI_LC_). VOI_RC_ and VOI_LC_ were independently processed by simple morphological closing using a z-axis elongated elliptical structure element to account for the cylindrical shape of the fascia and the predominant extension of the muscles along the leg axis, and contour-filling.

2. The processed VOI_RC_ and VOI_LC_ exclude the dermis, connective tissue and genital organs, whose CT values are typically similar to those of muscle. Within each of these VOIs, a simple contrast value threshold was used to identify probable muscle voxels (Fig 3c). These voxels were compacted (Fig 3d and 3e) resulting in a structure named VOI_IFA_, an approximation of the intrafascia VOI. Its surface was named fascia approximation (FA).

3. FA was already a very good approximation of the fascia when the amount of IMAT was low, i.e. in younger healthy subjects, in which the fascia is usually in direct contact with the muscle. However, with increasing adipose tissue infiltration, the amount of IMAT increases and the fascia is often bordered by adipose tissue on either side, SAT on the outer and IMAT on the inner side. In many areas, the CT contrast of the fascia is very low, preventing the direct detection of a closed surface. Instead, in our study FA was used as a start point to select additional voxels, which were likely to belong to VOI_IF_. Specifically, voxels, which were connected to FA, were not connected with the dermis and were located at a maximal distance of 15 voxels from FA, were identified as candidates (Fig 3f and 3g). Connection to the dermis was defined as voxels with C > 1 connected with the thigh surface. Based on volume growing, connection to FA was ensured by a local noise adaptive threshold. Namely, a voxel v was accepted by the volume growing if f was true:

$$\text{f}=\{\begin{array}{c} \text{true if C}>1\\ \text{g if }1\geq \text{C}\geq 0.5\\ \text{false else} \end{array}$$

where g depended on the mean CT value (mean), and the standard deviation (SD) in the 26-neighborhood of v:

$$\text{g}=\{\begin{array}{c} \text{true if CTvalue}\geq {\text{mean}}_{26}-2{\text{SD}}_{26}\\ \text{false else} \end{array}$$

**Fig 4:**
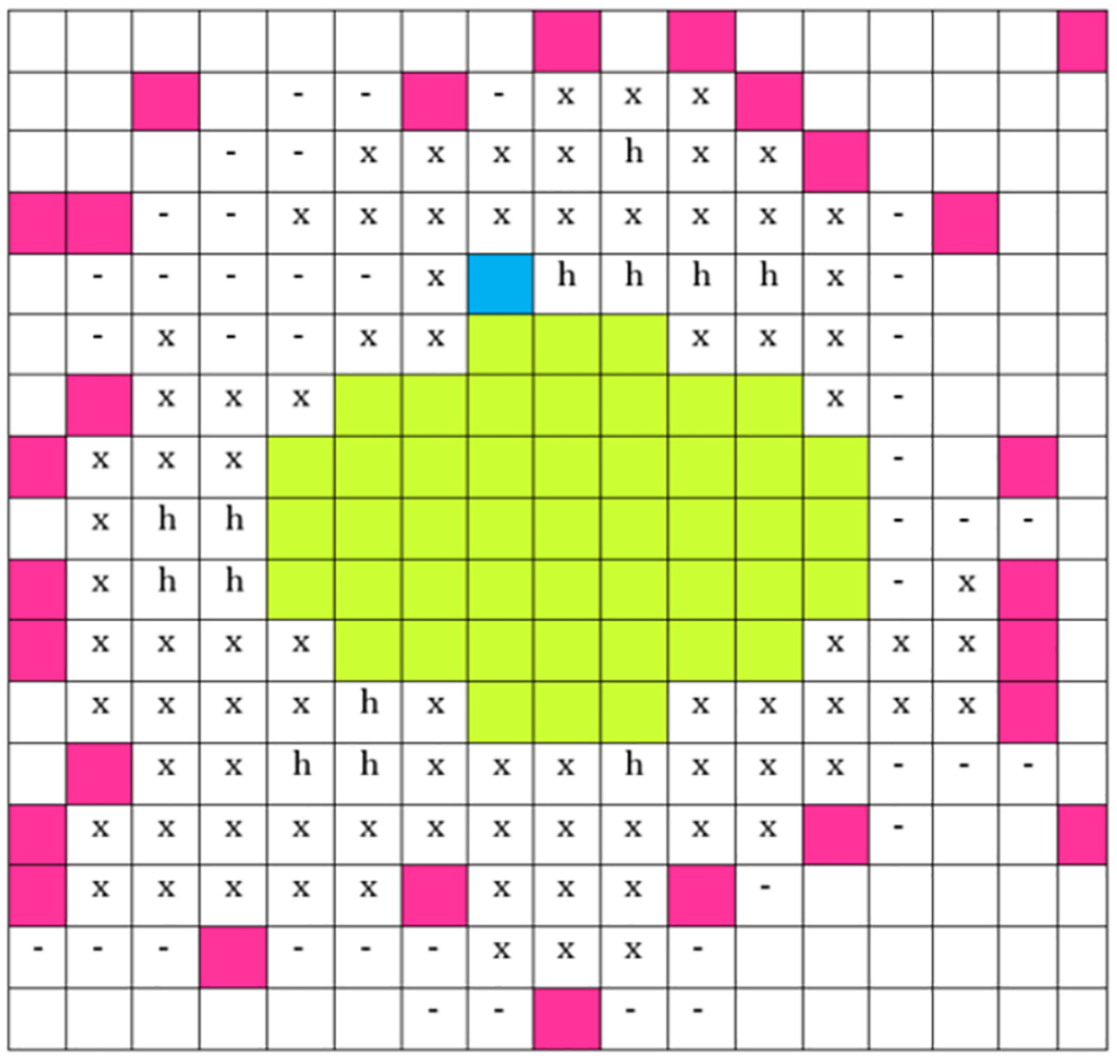
3D ray reflection model. **Approximation of the intrafascia VOI** (yellow), candidate voxels (purple) and seed point (blue) for the ray reflection model: The space between IFA and candidate voxels that were connected with the seed were either x: shielded, -: not-shielded or h: filled by contour-filling.

The 2SD factor accounted for noise in the CT data. Next, starting from a seed point set by the operator in the space between FA and a local group of the new candidate voxels, ‘rays’ were sent out isotropically (26 directions). If 65% or more of these rays were reflected by one of the candidate voxels or by FA, the status of the voxel was changed to ‘shielded’ [18] (Fig 4). All shielded and candidate voxels were merged with VOI_IFA_ (Fig 3i), which was then filtered for porous or non-compact structures by a morphological smoothing. The resulting VOI was called intrafascia (VOI_IF_, Fig 3j), its surface defined the 3D fascia. As shown in Fig 3h, often multiple seed points were required to process the complete dataset. The operator could also select not to add certain conglomerates of candidate voxels to VOI_IF_.

4. Optionally, a fascia-specific modification of the explicit deformable model by Mastmeyer [19] could be utilized to attract the fascia to the nearest maximum under constraint of a given regularity of the 3D surface. This step was useful, if neither strong edema nor very washed-out muscle structures were present in the image, which was typically the case for younger patients.

### C. Muscle and lipid VOIs

In order to facilitate a comprehensive analysis of the relation between muscle and lipids, various VOIs were determined. With the exception of VOI_SAT_, all of them were located within VOI_IF_. Specifically an anatomical muscle VOI_M_ and a muscle tissue VOI_MT_ were defined.

**Fig 5:**
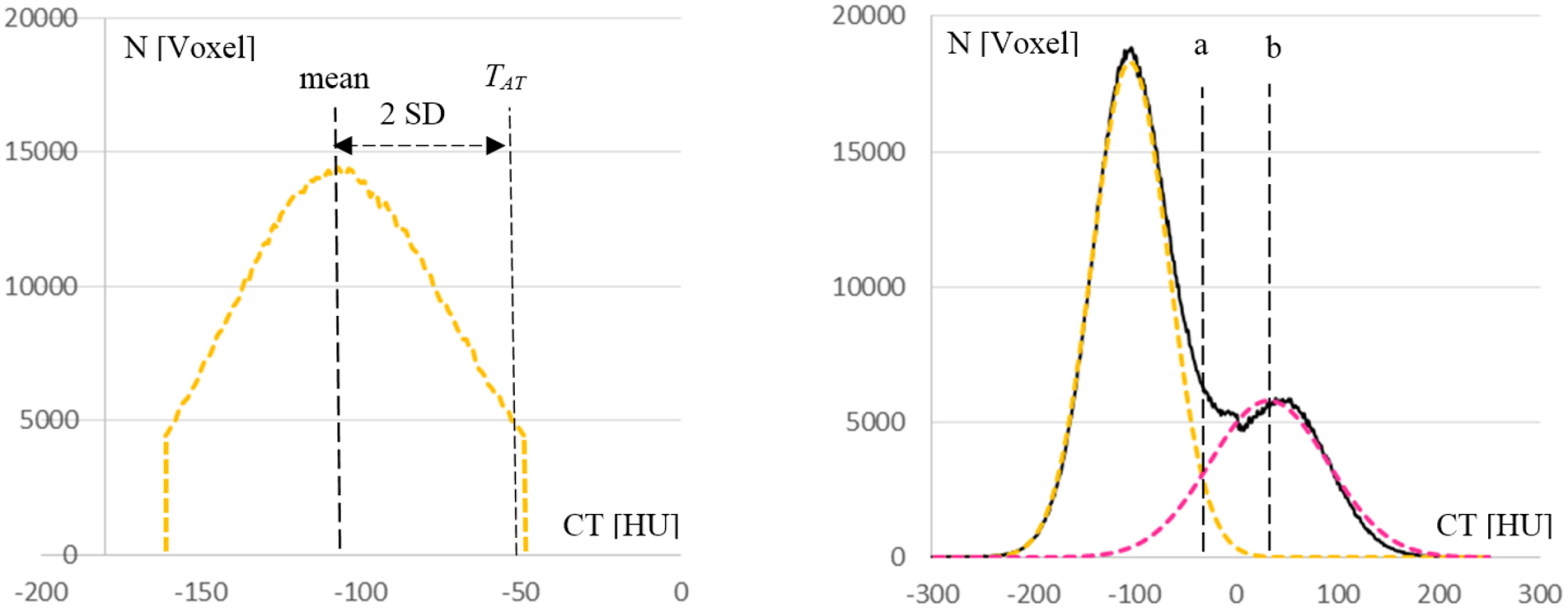
Image-specific definition of thresholds for anatomical muscle and muscle tissue. Left graph: definition of the adipose tissue threshold T_AT_ from SAT*; SD: standard deviation. Right graph: CT value spectrum of the combined subcutaneous adipose tissue and muscle tissue VOIs (VOI_MLS_) (black). Gaussian mixture model used to fit adipose (yellow) and muscle tissue (magenta) distributions; a: intersection between the two curves, b: peak of the muscle tissue curve.

1. VOI_SAT_ was obtained by subtracting VOI_IF_ and all voxels connected with the dermis from VOI_US_. The spectrum of CT values of VOI_SAT_ (Fig 5) was used to define an AT threshold (T_AT_), which differed from CT_AT_ used for the fascia segmentation. Typically, SAT is very homogenous but may contain edema and blood vessels that have higher CT values than adipose tissue. Therefore high and low CT values of the spectrum with frequencies lower than 30% of the most frequent CT value were cut. In order to determine T_AT_ from a symmetric spectrum to prevent an influence of noise on the mean value, the lower tail of the spectrum was also cut resulting in a spectrum SAT*. T_AT_ was defined as mean + 2SD from the mean and the standard deviation of the SAT* CT value histogram (Fig 5 left).

2. VOI_M_ was determined by subtracting VOI_PAT_ from VOI_IF_ under the assumption that PAT but not EML was connected with SAT. VOI_PAT_ was defined by connection with the fascia under the condition that the CT value of the voxel under consideration was smaller than T_AT_.

3. For the definition of VOI_MT_, a Gaussian mixture model (GMM) was employed in combination with a Levenberg-Marquard optimization algorithm [20] to fit two Gaussian curves to the CT value spectrum of the combined SAT and IF VOIs (VOI_SAT_ ∪ VOI_IF_ = VOI_MLS_) (Fig 5, right). One fit curve represented AT and one MT. For AT, height, peak and width of the SAT* distribution were used for initialization of the fit procedure. For MT the ratio of the number of voxels of VOI_AT_ versus those of VOI_MLS_ was used to initialize the height. A phantom based CT value was used to initialize its peak. VOI_MT_ was then defined by 3D volume growing inside VOI_IF_ starting from seed points defined as voxels with CT values higher than the peak b of the fitted MT curve. Voxels were included if their CT values were higher than that of the intersection a between AT and MT distributions (Fig 5, right).

4. Finally, in order to exploit the fact that the CT values of MT also reflect IML, similar to [21] an abstract description for the muscle-lipid distribution was formulated. For each voxel of VOI_IF_, a muscle concentration between 0% and 100% was determined using T_AT_ and T_HDM_ with T_AT_ defining a muscle concentration of 0% and T_HDM_, a muscle concentration of 100% (HDM: high density muscle). It was determined as mean CT value minus the SD determined in a sphere placed in the muscle tissue of young climbers. Results from 30 subjects were averaged and resulted in a CT value of 35 HU. In order to account for calibration differences among scanners, T_HDM_ was finally defined as 35 HU + CT_H20_. Thus, apart from the constant of 35HU, it only depended on the water value of the in-scan calibration phantom. The two CT value thresholds T_HDM_ and T_AT_ were used to define 6 bins with decreasing “muscle concentrations”. Bin 6 (B6) defined HDM for ≥ 100% muscle concentration, B5 muscle concentration for 75–99%, B4 for 50–74%, B3 for 25–49%, B2 for 0–24% and intermuscular adipose tissue (IMAT) for ≤ 0%.

### D. Feature extraction

Numerous image features can be calculated in the segmented VOIs to describe the muscle-lipid distribution. Only few features quantifying volume, density and structure will be discussed in the present report to investigate the reproducibility and stability of the segmentation and the feature extraction.

Densities were determined either from the segmentation-based or from GMM-based procedures. Segmentation-based densities were defined as average CT values in a VOI after subtraction of CT_H2O_. GMM-based densities were defined as peaks of IF or MLS distribution curves again after subtraction of CT_H2O_ i.e. D_IF_^GMM^(MT) or D_MLS_^GMM^ (MT). rV defined a volume of a VOI relative to VOI_MLS_ or VOI_IF_.

A more advanced 3D descriptor was the average grain size G_avg_ as determined by granulometry [22]. Porous and atrophic MT and HDM should have a smaller G_avg_. Fractal dimension (FD) [23] was used as a measure of 3D texture roughness. The 3D texture of dystrophic muscles should have a rougher appearance. Further, sphericity Ψ of M and MT was quantified. Ψ should be smaller for irregular surfaces and elongated muscle shape in case of pathologies.

### E. Validation

Validation of the segmentation stability and features included determination of the intra- and inter-operator precision errors and the impact of noise and of CT_H2O_ derived from the in-scan calibration phantom.

25 datasets from elderly subjects (age: 75.6 ± 8.7) of the EFFECT study [30] with a washed-out and moth-eaten muscle appearance and high levels of IMAT and edema were processed. In these subjects, FA differed largely in most cases from the true fascia and an operator-interaction was needed in such cases. For the precision analysis, all 25 datasets were analyzed three times on different days by the same operator for intra-operator-precision and once by three different operators after an initial training for inter-operator-precision. Precision errors were calculated as CV_RMS_ values [24]. In order to assess the diagnostic value of a feature, the CV_RMS_ was compared to the biological variation of the 25 subjects measured as percentage coefficient of variation divided by a factor of 100 (CV_100_).

Gaussian noise was added to the CT datasets acquired at 120 kV with 170 mAs to simulate a lower exposure of 135 mAs. Finally, CT_H2O_ obtained from the in-scan calibration phantom was changed by ± 5 HU.

## Results

**Fig 6:**
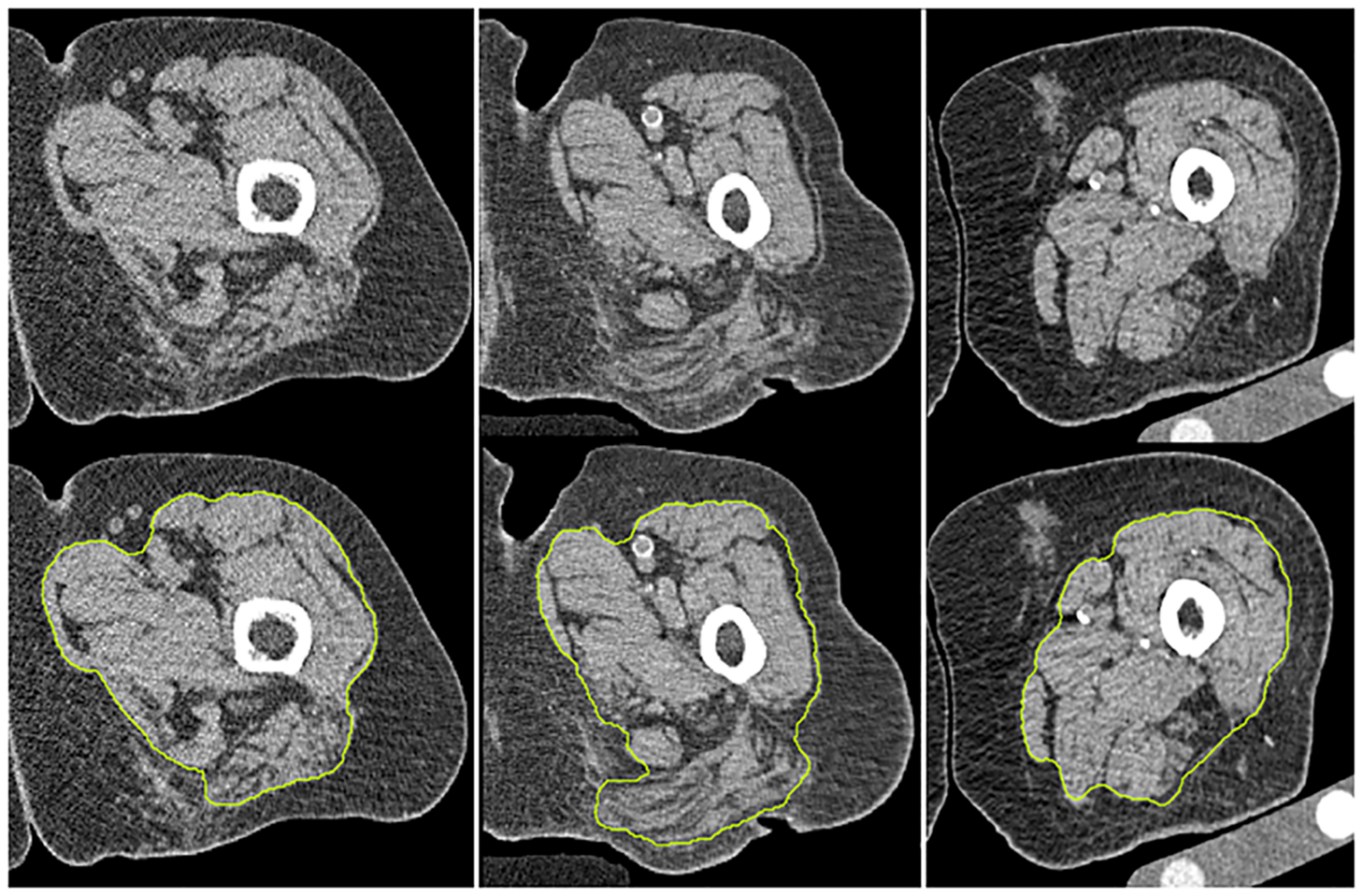
Segmentation results for three subjects with washed-out and moth-eaten muscles and strong edema. First row: native images; second row: segmented fascia (yellow).

Fig 6 shows segmentation results for three different patients, in which muscles were heavily infiltrated by adipose tissue and fascia not always in direct contact with the muscle. Thus, segmentation required operator interactions. However, even in these challenging cases the hierarchical segmentation process limited the required operator interaction to a few clicks by setting some seed points as shown in Fig 4f.

Depending on the segmentation complexity the complete processing time per CT dataset was around 12 min (i5 processor 5GHz, 4GB RAM). Table 1 shows results for intra- and inter-operator analysis precision errors. There were no repeat scans of patients. For comparison, Table 1 also shows the CV_100_ of the same feature from the 25 subjects. All CV_RMS_ results were below 2% and even below 0.6% in most cases reflecting the low impact of the operator interactions. Most of the CV_RMS_ results were comparable to the CV_100_ value.

**Table 1:** Reanalysis precision errors as CV_rms_ in per cent and biological variance as CV/100 in percent for density, relative volume and features for different VOIs. In each row intra/interoperator CV_rms_ results are on top and CV_100_ results below. D: density; D_If_^GMM^, D_MLS_^GMM^: density derived from Gaussian mixture model applied to of VOI_IF_ or VOI_MLS_, respectively; rV_MLS_, rV_IF_: Volume relative to VOI_IF_ or VOI_MLS_, respectively; G_avg_: average grain size; FD: fractal dimension; Ψ: sphericity. VOIs: SAT: subcutaneous adipose tissue; IF: intrafascia; M: muscle; MT: muscle tissue; HDM: high density muscle; IMAT: intermuscular adipose tissue. Note: not all features are determined for all VOIs.

	SAT	IF	M	MT	HDM	IMAT
D	0.06/0.10/	0.53 / 1.05	0.21 / 0.27	0.19 / 0.20	0.03 / 0.10	0.06 / 0.15
	0.07	0.79	0.27	0.23	0.08	0.18
D_IF_^GMM^				1.60 / 1.90		
				0.18		
D_MLS_^GMM^				0.53 / 0.56		
				0.44		
rV_IF_			0.11 / 0.24	0.10 / 0.27	0.20 / 0.33	0.51 / 1.90
			0.27	0.07	0.22	0.55
rV_MLS_	0.15 / 0.26	0.25 / 0.40				
	0.12	0.15				
G_avg_				0.24 / 1.1	0.04 / 0.15	0.18 / 0.31
				0.27	0.26	0.16
FD		0.03 / 0.05	0.04 / 0.05	0.05 / 0.06		
		0.017	0.019	0.021		
Ψ			0.57 / 0.62	0.55 / 0.70		
			0.20	0.23		

Table 2 shows the percentage changes after simulating an exposure level of 135 mAs, which would double the noise when compared to the 170 mAs exposure, which was actually used for the CT acquisition. Density and volume measurements of VOI_SAT_ and VOI_IF_ were not affected by higher noise. For most other features, the noise related change was about one magnitude higher than the reanalysis precision error at 170 mAs. Highest noise related changes were measured for the volume of VOI_IMAT_ relative to the volume of VOI_IF_ and for the average grain size of VOI_MT_

**Table 2:** Percentage changes for density, relative volume and features for different VOIs when an exposure level of 135 mAs was simulated. D: density; D_IF_^GMM^, D_MLS_^GMM^: density derived from Gaussian mixture model applied to of VOI_IF_ or VOI_MLS_, respectively; rV_MLS_, rV_IF_: Volume relative to VOI_IF_ or VOI_MLS_, respectively; G_avg_: average grain size; FD: fractal dimension; Ψ: sphericity. VOIs: SAT: subcutaneous adipose tissue; IF: intrafascia; M: muscle; MT: muscle tissue; HDM: high density muscle; IMAT: intermuscular adipose tissue.

	SAT	IF	M	MT	HDM	IMAT
D	0.0	0.0	5.1	3.5	3.4	2.6
D_IF_^GMM^				4.0		
D_MLS_^GMM^				4.2		
rV_MLS_	0.0	0.0				
rV_IF_			2.0	1.1	0.71	15.0
G_avg_				14.1	4.9	2.1
FD		0.89	0.79	0.99		
Ψ			3.8	8.1		

For the features or segmentations underneath the fascia that were calibrated by water calibration or more specifically CT_H2O_, minor variations (± 5 HU) in the CT_H2O_ value, which in our case was obtained from the in-scan calibration phantom, caused a much larger effect than a increase in noise. Apart from the density of HDM, a small change (± 5 HU) in the CT_H2O_ value caused density differences of more than 10% (Table 3) although relative to CV_100_ results were comparable.

**Table 3:** Effect of water calibraton. A simulated addition / subtraction of 5 HU from CT_H2O_.caused the percent change shown in the table. D: density; D_IF_^GMM^, D_MLS_^GMM^: density derived from Gaussian mixture model applied to of VOI_IF_ or VOI_MLS_, respectively; SAT: subcutaneous adipose tissue; IF: intrafascia; M: muscle; MT: muscle tissue; HDM: high density muscle muscle.

	IF	M	MT	HDM
D	27.4 / 27.4	15.8 / 15.8	13.4 / 13.4	3.2 / 3.5
D_IF_^GMM^			11.6 / 11.6	
D_MLS_^GMM^			17.8 / 17.8	

## Discussion

To our knowledge, this is the first study presenting a hierarchical 3D approach to segment and quantify the different muscular and lipid components of the thigh. One critical step is segmentation of the fascia, which separates SAT from IMAT and muscle from edema, blood vessels, genital organs etc. Calibration based on the relatively homogeneous subcutaneous adipose tissue and the CT value of water, which, in the present work, was determined using an in-scan calibration phantom, is another important characteristic of our method. The third critical step is the attempt to characterize muscle in different manners: as anatomical muscle, as muscle tissue, and in an abstract way—comparable to grey matter concentration in neuroimaging [25]—as muscle concentrations.

The most difficult step was the segmentation of the fascia covering the muscles, which, in elderly subjects, is no longer in tight contact with the muscle surface. The main purpose of most of the preparatory steps illustrated in Fig 3a–3i was the identification of a search space for voxels likely to belong to VOI_IF_. A second purpose was the differentiation of external genital organs, vessels, dermis and edema from the moth-eaten and washed out muscle as shown in Fig 3. CT value differences, for example between edema and washed out muscle structures, are small and therefore techniques based on a simple global threshold to separate muscle and adipose tissue will have severe limitations. Our method based on fascia segmentation and integration of anatomical knowledge does not have such inconvenients (Fig 6).

A limitation to our study is the lack of comparison to a gold standard such as manual segmentation. However, manual segmentation is highly fastidious and therefore almost not feasible in 3D and not used by radiologists who prefer to perform semi-quantitative grading based on gross muscle CT appearance [1–3]. We also have not applied our method to patients with diseases that largely destroy muscle tissue. In this case, the separation of adipose and muscle tissue as shown in Fig 5 may fail. Further, we have not analyzed any longitudinal data in order to quantify the effect of age, disease or treatment related morphological changes on segmentation. Finally, we have not analyzed the impact of spatial resolution on segmentation. CT images used here had a slice thickness of 1 mm and an in plane voxel size of (0.8 mm)^2^ and were reconstructed with a medium kernel. This provides an adequate balance between spatial resolution, noise and radiation exposure. Different settings of CT acquisition and reconstruction parameters would be required to evaluate the impact of spatial resolution.

The very low intra- and inter-operator precision errors of our method, below 1% (Table 1) is due to the high degree of automation of the analysis. Manual interactions as those shown in (Fig 3h) and pre-grading of muscle structures (Fig 3a and 3b) had minimal impact on precision. However, we did not carry out a precision study including patient repositioning, which may increase precision error, since muscle shape may change across multiple scans. Repeating CT acquisitions in humans is difficult due to ethical issues.

Precision error has to be put in perspective with the changes to be measured, either longitudinally to assess changes in a given subject or cross-sectionally to compare different subjects. Table 1 shows that the reanalysis precision errors were comparable with 1% of the variation of the corresponding measurement in 25 elderly female subjects, again a very good result.

It remains speculative whether other segmentation methods [26–29] would improve the results obtained here. When applying a hierarchical approach, selection of the different steps and their order remains subjective. To our knowledge, starting with a segmentation of the fascia, an anatomical boundary at the superficial aspect of the muscles is an innovative method. However, a comparison of the different segmentation methods remains to be done.

Existing techniques for muscle measurements on CT images are restricted to measure muscle volume or area and density. Several authors used fixed CT value thresholds for muscle segmentation [30–32]. Use of fixed thresholds to carry out muscle segmentation does not address differences in muscle density caused by variable degrees of muscle lipid infiltration, which vary widely among muscles.

Use of fixed CT values or Hounsfield units also does not address variation with scanner type and manufacturer. Although all clinical CT scanners are routinely calibrated to water, deviations of ±10 HU are frequent. In addition, a second value is required for calibration of muscle and adipose tissue. In the field of osteodensitometry [33], specific in-scan phantoms are used for the calibration of CT to measure bone mineral density.

In the method developed here, calibrated CT values were used for two different purposes. In the segmentation process CT_H2O_, the CT value of the water insert of the calibration phantom and the mean CT value of adipose tissue were used to define a contrast scale, which was primarily used to define probable muscle voxels and to define upper and lower limits for the local adaptive threshold based ray reflection model. However, the final fascia resulted from the combination of several advanced image-processing procedures rather than from simple thresholding.

For segmentation of VOIs inside the fascia, two CT values derived from image information were used, CT_H2O_ and a second CT value defined precisely using the SAT CT value histogram (Fig 6). SAT is one of the most homogenous adipose tissues in the human body [34], and is well-suited for this purpose but, in order to exclude higher density voxels due to vessels and edema contained into the SAT, the histogram was trimmed, which resulted in a unique subject-specific threshold T_AT_. Finally, the threshold for 100% muscle depended only on CT_H2O_ and a constant determined from muscle tissue of a group of young athletes.

Thus, our segmentation method uses a calibration that depends only on subject-specific internal CT values and CT_H2O_ and is therefore independent from the scanner model and manufacturer. An accurate determination of CT_H2O_ is critical: as shown in Table 3, a change of ± 5 HU in CT_H2O_ results in a change in density values of up to 30%, emphasizing that deviations from the regular water calibration of clinical CT scanners must be controlled.

Another critical characteristic of segmentation and feature extraction is the sensitivity to noise. To test our method, we simulated a 100% noise increase by retrospectively adding Gaussian noise to the CT images. Table 2 shows that density and volume measurements of SAT were not affected by a 100% noise increase, which is very important with regard to the calibration. With the exception of rV_IF_ of V_IMAT_, effects on muscle density and volume measurements were below 5%. Not surprisingly, an increase in noise had larger effects on structural measurements like 3D texture features. Automatic exposure control techniques, nowadays often used in clinical CT [35], can approximately guarantee similar noise levels between patients. A new data-driven method to identify and reduce the impact of technical variation—such as noise—on features is currently developed in our group for e.g. retrospective analyses of studies without automatic exposure control techniques.

## Conclusion

We have developed an innovative semi-automatic approach for segmentation and quantification of the muscle-lipid distribution in CT images of the thigh. An important characteristic is the calibration combining subject-specific internal subcutaneous adipose tissue with an externally derived water calibration. Another characteristic is the hierarchical segmentation of the fascia consisting of a pre-grading based on established diagnostic criteria and a local adaptive threshold-based 3D ray reflection model which requires only minimal user interaction and offers an automatic refinement by an explicit deformable model. Extracted features included density- and volume-based muscle and lipid measurements as well as advanced 3D features for a detailed quantification of the muscle-lipid distribution underneath the fascia. The precision of the method was excellent and processing speed acceptable, enabling a comprehensive radiomics approach to musculoskeletal lipid distribution disorders from standard-of-care CT images. Results reported here concern the thigh but the same concept may be applicable to other body parts although some anatomy specific changes will be required in the segmentation step.
